# STRESS-BUFFERING EFFECTS OF VOLUNTEERING ON DAILY WELL-BEING: RESULTS FROM THE NATIONAL STUDY OF DAILY EXPERIENCES

**DOI:** 10.1093/geroni/igz038.876

**Published:** 2019-11-08

**Authors:** Saehwang Han, Kyungmin Kim, Jeffrey Burr

**Affiliations:** 1 University of Texas at Austin, Austin, Texas, United States; 2 University of Massachusetts Boston, Boston, Massachusetts, United States

## Abstract

Based on theory and empirical evidence linking volunteering and health, we investigated the associations between daily engagements in formal volunteering, stressors, and negative affective well-being, focusing on the stress-buffering effect of volunteering. Using eight days of daily diary data from the second wave of the National Study of Daily Experiences (participants, N = 1,320; participant-day observations, N = 8,277), we estimated a series of multilevel models to assess the within-person associations between daily volunteering, stressors, and affect. Results indicated there were no direct associations between daily volunteering and negative affect. However, we found the association between daily stressors and negative affect (but not positive affect) was weaker on days when volunteering was performed compared to days volunteering was not performed. Taken together, our findings suggested that short-term health benefits associated with daily volunteering were largely based on the stress-buffering effects of helping others, rather than through a direct effect.

